# Cytokinin activity increases stomatal density and transpiration rate in tomato

**DOI:** 10.1093/jxb/erw398

**Published:** 2016-11-02

**Authors:** Mika Farber, Ziv Attia, David Weiss

**Affiliations:** Institute of Plant Sciences and Genetics in Agriculture, The Robert H. Smith Faculty of Agriculture, Food and Environment, The Hebrew University of Jerusalem, P.O. Box 12, Rehovot 76100, Israel

**Keywords:** Cytokinin, drought, stomata, stomatal density, tomato, transpiration.

## Abstract

Previous studies on cytokinin (CK) and drought have suggested that the hormone has positive and negative effects on plant adaptation to restrictive conditions. This study examined the effect of CK on transpiration, stomatal activity, and response to drought in tomato (*Solanum lycopersicum*) plants. Transgenic tomato plants overexpressing the *Arabidopsis thaliana* CK-degrading enzyme CK oxidase/dehydrogenase 3 (CKX3) maintained higher leaf water status under drought conditions due to reduced whole-plant transpiration. The reduced transpiration could be attributed to smaller leaf area and reduced stomatal density. CKX3-overexpressing plants contained fewer and larger pavement cells and fewer stomata per leaf area than wild-type plants. In addition, wild-type leaves treated with CK exhibited enhanced transpiration and had more pavement cells and increased numbers of stomata per leaf area than untreated leaves. Manipulation of CK levels did not affect stomatal movement or abscisic acid-induced stomatal closure. Moreover, we found no correlation between stomatal aperture and the activity of the CK-induced promoter *Two-Component Signaling Sensor* (*TCS*) in guard cells. Previous studies have shown that drought reduces CK levels, and we propose this to be a mechanism of adaptation to water deficiency: the reduced CK levels suppress growth and reduce stomatal density, both of which reduce transpiration, thereby increasing tolerance to prolonged drought conditions.

## Highlight

Manipulation of cytokinin levels in tomato plants affected transpiration rate. While the hormone had no effect on stomatal movement, it increased stomatal density by promoting epidermal cell division.

## Introduction

Under natural conditions, plants are often exposed to a range of biotic and abiotic stresses, which affect their growth and development (Farooq *et al.*, 2009, 2012; Seki *et al.*, 2003). Among the abiotic stresses, drought is one of the most devastating (Boyer, 1982); it affects flora biodiversity and causes major losses in crop productivity (Shao *et al.*, 2009). Plants can adjust to drought to a limited extent through rapid physiological responses (stomatal closure) and phenotypic plasticity (Chaves *et al.*, 2003; Farooq *et al.*, 2009; Osakabe *et al.*, 2014). Some of these responses are mediated by phytohormones. Both rapid and developmental responses to drought are mediated primarily by the stress-related hormone abscisic acid (ABA; Zhu, 2002). However, growth-promoting hormones, such as gibberellins (GAs) and cytokinins (CKs), are also involved in the response to drought (Achard *et al.*, 2006; Ha *et al.*, 2012; Nir *et al.*, 2014). Thus, it seems that the balance between stress-related and growth-promoting hormones has an influence on plant performance during short episodes of drought and on their adaptation to prolonged drought conditions.

CK is a major growth-promoting hormone that regulates various developmental processes, including cell division and senescence (Schaller *et al.*, 2014). Studies of CK sensing and signaling, using molecular genetic approaches, have led to the discovery of major positive and negative signaling components. CKs bind to the His-kinase receptors and induce their autophosphorylation. His-phosphotransfer proteins transfer the phosphate group from the receptor to the nucleus to phosphorylate and activate a set of transcription factors known as type-B response regulators (RRs or, in Arabidopsis, ARR). The activated type-B RRs induce the transcription of various CK-regulated genes, including genes encoding type-A RRs, which in turn suppress CK responses (Hwang *et al.*, 2012).

The role of CK in plant adaptation to environmental stresses, including drought, has been studied intensively in the past two decades (O’Brien and Benková, 2013; Pospíšilová *et al.*, 2000; Zwack and Rashotte, 2015). Nevertheless, it is still not clear whether CK promotes or suppresses drought tolerance (Zwack and Rashotte, 2015). Nishiyama *et al.* (2011) showed that Arabidopsis mutated in the central CK-biosynthesis genes *ISOPENTENYL TRANSFERASES* (*IPT*s) exhibit better survival rates under drought stress. Similarly, Arabidopsis mutants in CK-signaling components—His-kinase receptors, His phosphotransfer proteins, and type-B RRs—exhibit increased tolerance to drought (Kang *et al.*, 2012; Kumar and Verslues, 2015; Nguyen *et al.*, 2016; Nishiyama *et al.*, 2011, 2013; Tran *et al.*, 2007). On the other hand, Kang *et al.* (2012) reported that Arabidopsis plants grown on CK-containing media are less sensitive to drought, exhibiting higher survival rates under these conditions. Moreover, overexpression of *IPT* in several plant species improves plant performance under drought conditions (Merewitz *et al.*, 2012; Peleg *et al.*, 2011; Rivero *et al.*, 2007). Several mechanisms have been proposed to explain the positive and negative effects of CKs, including effects on membrane integrity, sensitivity to ABA (Kang *et al.*, 2013; Nishiyama *et al.*, 2011), proline accumulation (Kumar and Verslues, 2015), maintenance of photosynthetic activity (Rivero *et al.*, 2009, 2010), regulation of sink/source activity (Reguera *et al.*, 2013), and regulation of root architecture (Werner *et al.*, 2010).

Other studies have examined how CK affects water balance and whether it regulates stomatal movement. Application of low concentrations of CK to *Vicia faba* abaxial epidermal peels promoted stomatal opening in the dark, whereas high concentrations had no effect (Morsucci *et al.*, 1991; Song *et al.*, 2006). In Arabidopsis, CK treatment had no effect on stomatal aperture, but it inhibited ABA-induced stomatal closure (Tanaka *et al.*, 2006). Overexpression of *IPT* under the regulation of the *RUBISCO SMALL SUBUNIT* (*PSSU*) promoter in tobacco increased transpiration rate in young developing leaves but decreased it in older leaves (Synková *et al.*, 1999). Rivero *et al.* (2009) and Reguera *et al.* (2013) expressed *IPT* under the regulation of the *SENESENCE ASSOCIATED RECEPTOR KINASE* (*SARK*) promoter in tobacco and rice, respectively. Their results suggested that elevated levels of CK during drought-induced senescence maintain relatively high stomatal conductance and inhibit the decrease in transpiration. In contrast, CK induced stomatal closure in *Commelina* plants grown in the light (Blackman and Davies, 1983). Finally, overexpression of the bacterial *ZMP* gene, which encodes an enzyme that converts inactive CK conjugates such as CK-O-glucosides to active CKs, reduced transpiration in young leaves, but increased it in mature leaves (Pospíšilová *et al.*, 1997). Taking these findings together, CK seems to have opposite effects on leaf transpiration in different plant species, and even in different leaves on the same plant.

In this study, we examined how CK affects transpiration in tomato (*Solanum lycopersicum*) plants and studied the underlying mechanism. Our results suggest that CK promotes transpiration indirectly, by increasing stomatal density. Accordingly, reduced CK levels suppress transpiration by reducing stomatal density, but also by suppressing growth and promoting leaf senescence.

## Materials and methods

### Plant material

Tomato (*S. lycopersicum*) plants used in this study were in the M82, *SP* ^*–*^ background. The plants were grown in a growth room under an 11/13 h day/night photoperiod at 25 °C or in a semi-controlled greenhouse under natural day length and light conditions at an average temperature of 26 °C.

### Hormone treatments

All exogenous applications of the synthetic CK 6-benzylaminopurine (BA) (Sigma-Aldrich, St Louis, MO, USA) were performed by either spraying or immersing the plants for 5 min. ABA (Sigma-Aldrich) was applied by spraying. All hormone treatments included the surfactant Tween 20 (100 µl l^−1^).

### Chlorophyll extraction and measurements

Chlorophyll was extracted from fresh leaves in 100% acetone and measured spectrophotometrically at 645 and 663 nm (Arnon, 1949). Chlorophyll concentration was calculated using the formula:

$$\text{Chlorophyll concentration }\left({\text{mg ml}}^{–\text{1}}\right)=\left(\text{2}0.\text{2}\times {\text{A}}_{\text{645}}\right)+\left(\text{8}.0\text{2}\times {\text{A}}_{\text{663}}\right).$$

### Leaf relative water content measurement

Detached leaves were weighed (fresh weight [FW]) and then immersed in 5 mM CaCl_2_ solution for 8 h. The leaves were weighed again at full turgidity (turgid weight [TW]) and then stored at 60 °C for 48 h. Leaf dry weight (DW) was then recorded, and leaf relative water content (RWC) was calculated according to Barrs and Weatherley (1962):

$$RWC\left(\%\right)=\frac{\left(FW-DW\right)}{\left(TW-DW\right)}\times 100$$

### Whole-plant transpiration and stomatal conductance measurements

Whole-plant transpiration rates were determined using an array of lysimeters placed in the greenhouse, as described in detail in Sade *et al.* (2009). Briefly, control M82 and transgenic plants were planted in 3.9 L pots and grown under semi-controlled conditions (30/18 °C day/night under natural day length and light intensity ~500 μmol m^−2^ s^−1^). Each pot was placed on a temperature-compensated load cell with digital output (Vishay Tedea-Huntleigh, Netanya, Israel) and sealed to prevent evaporation from the surface of the growth medium. The weight output of the load cells was monitored every 10 s and the average readings over 3 min were logged in a model CR1000 data logger (Campbell Scientific, Logan, UT, USA) for further analysis. Plant daily transpiration rate (weight loss between predawn and sunset) was calculated from the weight difference between the two data points. Stomatal conductance value, *g*_s_ (mmol m^−2^ s^−1^), was calculated from the transpiration rate and vapor-pressure deficit (VPD) data. VPD was calculated from the temperature and humidity values measured in the greenhouse (Buck, 1981; Farquhar, 1978). Plant daily transpiration rate and stomatal conductance were normalized to total leaf area, which was measured at the end of the experiment using a model Li 3100 leaf area meter (Li-Cor, Licor Biosciences, Lincoln, NE, USA).

### Relative soil volumetric water content measurements

Relative soil volumetric water content (VWC) was measured using a model EC-5 soil moisture sensor (Decagon Devices, Pullman, WA, USA).

### Drought stress treatment

Drought stress was applied by withholding irrigation until VWC reached 10%.

### Microscopic analyses of stomatal aperture, density, and index

Stomatal aperture and density were determined using the rapid imprinting technique (Geisler *et al.*, 2000). This approach allowed us to reliably score hundreds of stomata from each experiment simultaneously. Briefly, light-body vinylpolysiloxane dental resin (eliteHD+, Zhermack Clinical, Badia Polesine, Italy) was attached to the abaxial side of the leaf, dried for ~1 min and then removed. The resin epidermal imprints were covered with transparent nail polish, which was removed once it had dried and served as a mirror image of the resin imprint. The nail-polish imprints were placed on glass cover slips and photographed under a model 1M7100 bright-field inverted microscope (Zeiss, Jena, Germany) with a mounted HV-D30 CCD camera (Hitachi, Japan). Stomatal images were later analyzed to determine aperture size using the ImageJ software (http://rsb.info.nih.gov/ij/) fit-line tool. A microscopic ruler (Olympus) was used for size calibration.

### Transpiration measurements

Transpiration rate was measured with a model SC-1 steady-state diffusion porometer (Decagon Devices, Pullman, WA, USA) and calculated from *g*_S_ data. The porometer also measures air temperature (T) and air relative humidity (RH), from which the VPD values were calculated (Buck, 1981). From the *g*_S_ and VPD values, transpiration rate (E) was calculated, following Farquhar (1978), as:

$$E=\frac{g_{s}\times VPD}{P_{atm}}$$

### Stomatal aperture measurements in detached epidermis

Abaxial epidermal strips were peeled and the detached layers were incubated in stomatal opening buffer (Wigoda *et al.*, 2006) for 2 h. The strips were then placed on glass cover slips and photographed under the bright-field inverted microscope as described above. Stomatal images were later analyzed to determine aperture size using the ImageJ software fit-line tool. A microscopic ruler was used for size calibration.

### RNA extraction, cDNA synthesis and quantitative reverse transcription-PCR analysis

Total RNA was isolated from leaves of 4-week-old tomato plants. RNA extraction and cDNA synthesis were performed as described by Livne *et al.* (2015). Quantitative reverse transcription-PCR analysis was performed using the Absolute Blue qPCR SYBR Green ROX Mix (AB-4162/B) kit (Thermo Fisher Scientific). Reactions were performed using a Rotor-Gene 6000 cycler (Corbett Research). A standard curve was obtained for each gene using dilutions of a cDNA sample. Each gene was quantified using Corbett Research Rotor-Gene software. At least three independent technical repeats were performed for each cDNA sample. The relative expression of each sample was calculated by dividing the expression level of the analyzed gene by that of *TUBULIN*. Gene-to-*TUBULIN* ratios were then averaged. All primer sequences are presented in Supplementary Table S1 at *JXB* online.

### Constructs and plant transformation

The sequence of the synthetic promoter *Two-Component Signaling Sensor* (*TCS*) version 2 (Steiner *et al.*, 2016) was ligated to the β-galactosidase (GUS) gene from *Escherichia coli* to generate a *TCSv2:GUS* fusion in pART27. This construct was introduced into *Agrobacterium tumefaciens* and then transferred to M82 cotyledons using the transformation and regeneration methods described by McCormick (1991). Kanamycin-resistant transformants were selected as described previously (Shani *et al.*, 2009).

### Transactivation system

The LhG4 transactivation system (Moore *et al.*, 1998) consists of a driver line that expresses the synthetic transcription factor *LhG4* under the control of a specific promoter (promoter:LhG4), and a responder line in which the target gene is expressed under an operator array (OP) from *E. coli*. This operator array is recognized by LhG4, and therefore crossing a driver line with a responder line leads to expression of the target gene under the specific promoter in the progeny. To express the Arabidopsis *CK OXIDASE/DEHYDROGENASE3* gene (*AtCKX3*) (Bilyeu *et al.*, 2001) under the regulation of the 35S promoter, we used *35S:LhG4* as the driver line (Alvarez *et al.*, 2006) and *OP:AtCKX3* as the responder line (Shani *et al.*, 2010) and crossed them to generate the transactivated line *35S>>AtCKX3* (Shani *et al.* 2010).

### Histochemical GUS analysis

For histochemical analysis of GUS activity, leaves of 2-week-old tomato *TCSv2:GUS* plants were collected, placed in cold acetone (70%) and vacuum-infiltrated for 5 min. Detection was then performed using 5-bromo-4-chloro-3-indolyl β-D-glucuronide as described previously (Donnelly *et al.*, 1999). Samples were imaged after immersing the leaves in 50% glycerol and then placing them on a microscope slide and photographing them using a model SMZ1500 fluorescence stereomicroscope (Nikon Instruments Inc., Melville, NY, USA) equipped with a Nuance camera (Cri) (Quorum Technologies Inc., Guelph, Ontario, Canada).

### Yellow fluorescent protein signal analyses

Analyses of the yellow fluorescent protein (YFP) signal in guard cells was performed using a model SP8 confocal laser-scanning microscope (Leica Microsystems, Herzliya, Israel). The laser was set at 514 nm excitation and 520–560 nm emission. To detect the YFP signal, the abaxial epidermis was peeled from the leaf of *TCSv2:3XVenus* (YFP) plants (provided by Professor Yuval Eshed, Weizmann Institute of Science), then placed on a microscope slide and immersed in stomatal opening buffer (as described by Wigoda *et al.*, 2006). Signal intensity was determined using LAS AF Lite 2.6.3 software (Leica Microsystems).

### Statistical analyses

All assays were conducted with three or more biological replicates and analyzed using JMP software (SAS Institute, Cary, NC, USA). Mean comparisons were conducted using Tukey–Kramer HSD and Student’s *t* tests, with significance accepted at *P*<0.05.

## Results

### Reduced levels of CK decrease whole-plant transpiration

To determine how changes in endogenous levels of CKs affect transpiration, we generated transgenic (transactivated) tomato plants overexpressing *AtCKX3* under the regulation of the 35S promoter (*35S>>CKX3*). To this end, we used *35S:LhG4* as the driver line (Alvarez *et al.*, 2006) and *OP:AtCKX3* as the responder line (Shani *et al.*, 2010) and crossed them to generate the transactivated line *35S>>AtCKX3*. This transactivated line was described previously by Shani *et al.* (2010). It should be noted that both driver and responder lines have a wild-type phenotype (Alvarez *et al.*, 2006; Shani *et al.*, 2010). Quantitative reverse transcription-PCR analysis revealed a high level of *AtCKX3* expression in the transactivated leaves (Supplementary Fig. S1A). The *35S>>CKX3* plants displayed a typical CK-deficiency phenotype, including slow and retarded growth rate and simple leaves (Supplementary Fig. S1; Shani *et al.*, 2010; Werner *et al.*, 2003). To confirm that these phenotypic changes resulted from reduced levels of active CKs, we treated the transgenic plant with BA, a synthetic CK that is resistant to CKX (Gerhauser and Bopp, 1990). BA application restored normal leaf complexity but had only a mild effect on leaf size (Supplementary Fig. S2). It has previously been shown that the application of exogenous BA cannot mimic the effect of endogenous CKs on tomato leaf development (Fleishon *et al.*, 2011; Shwartz *et al.*, 2016).

**Fig. 1.**
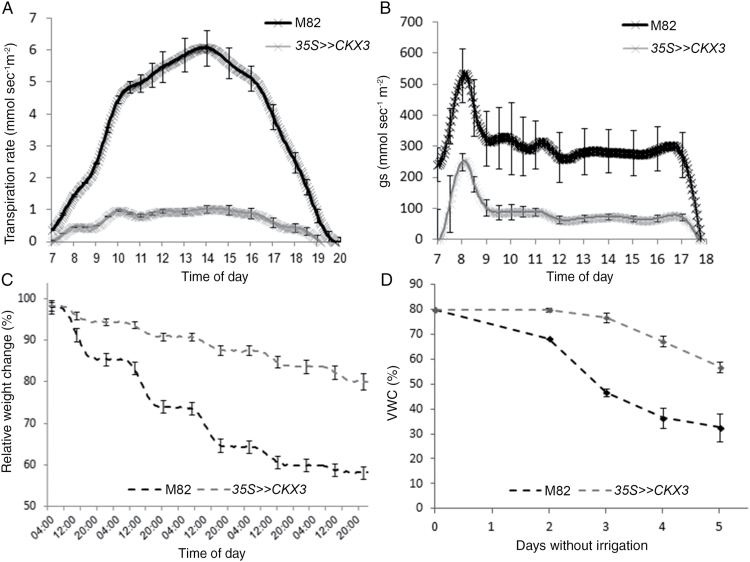
*AtCKX3* overexpression reduces whole-plant transpiration under irrigation and during drought stress in tomato. One-month-old *35S>>CKX3* and M82 plants were placed on the lysimeter system and, after 2 weeks, irrigation was halted. (A) Transpiration rate under normal irrigation normalized to total leaf area. (B) Stomatal conductance under normal irrigation normalized to total leaf area. (C) Pot (pot+soil+plant) weight was measured for 5 days without irrigation. Pot weight was measured every 10 s and readings were averaged over 3 min. For clarity, SE bars are shown for only three time points during each 24 h cycle. (D) Soil relative VWC in pots of *35S>>CKX3* and M82 plants during 5 days without irrigation. Values are means±SE of six replicates.

**Fig. 2.**
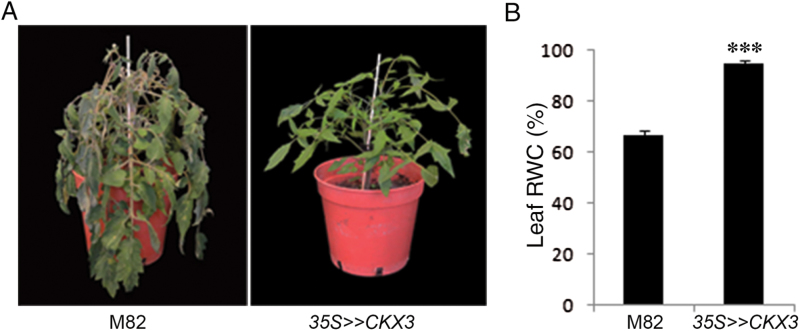
*AtCKX3* overexpression reduces water loss in tomato. M82 and *35S>>CKX3* plants were grown for 2 months and then irrigation was stopped to produce dehydration. Phenotype and RWC were recorded after 4 days. (A) M82 and *35S>>CKX3* plants 4 days after the beginning of the drought treatment. (B) RWC in leaf number 3 (counted from the bottom of the plant up). Values are means±SE of five biological replicates. (This figure is available in colour at *JXB* online.)

Next, we analyzed transpiration in the transgenic plants under normal and drought conditions. To measure whole-plant transpiration, we used an array of load cells (lysimeters; see Materials and Methods) placed in the greenhouse and simultaneously measured the daily weight loss of each plant. Under a normal irrigation regime, the daily transpiration rate (normalized to leaf area to eliminate the effect of plant size) of the *35S>>CKX3* plants was significantly lower than that measured for M82 plants (Fig. 1A). The reduced endogenous level of CKs did not, however, affect the pattern of daily transpiration, suggesting a similar pattern of stomatal movement (Fig. 1B, Supplementary Fig. S3). After 5 days of water deprivation, M82 plants reached a VWC of 33%, while at the same time the *35S>>CKX3* plants reached 57% (Fig. 1D). As a result, at this time point, control plants wilted, whereas the *35S>>CKX3* plants maintained high RWC and were fully turgid (Fig. 2). The transgenic plants maintained their low and stable transpiration rate throughout the drought treatment (Fig. 1C) and reached 30% VWC only after 7 days of drought. Thus, the reduced transpiration in the *35S>>CKX3* plants led to slower use of the available water in the soil.

**Fig. 3.**
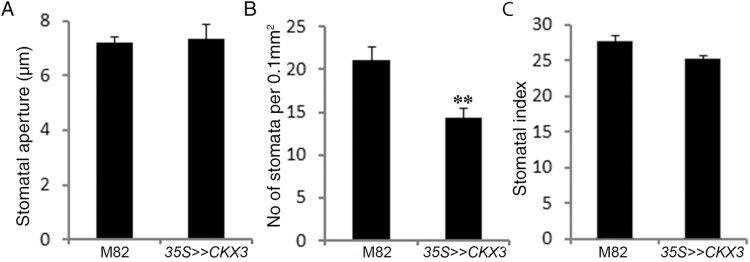
*AtCKX3* overexpression reduces stomatal density in tomato. Imprints of abaxial epidermis were taken from leaf number 3 (from the bottom up) of 6-week-old *35S>>CKX3* and M82 plants. (A) Stomatal aperture measured at 10.00 h. (B) Number of stomata per 0.1 mm^2^. (C) Stomatal index (stomata:epidermal cell ratio). Values are means±SE of four replicates. Each replicate is the average of ~100 measurements (stomata). ** Significant difference (Student’s *t* test; *P*<0.01).

**Fig. 4.**
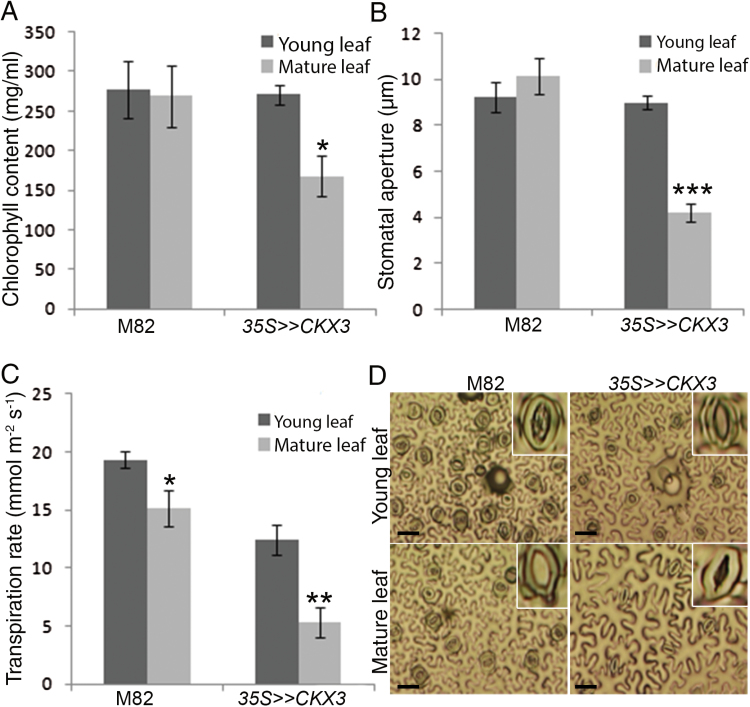
*AtCKX3* overexpression accelerates leaf senescence and reduces transpiration in tomato. (A) Chlorophyll content. Values are means±SE of five replicates. (B) Stomatal aperture measured on imprints of abaxial epidermis taken at 11.00 h. Values are means±SE of four replicates, each with ~100 measurements (stomata). (C) Transpiration rate calculated from stomatal conductance measured at 10.00 h using a Decagon SC-1 leaf porometer. In (A) to (C), all measurements were taken from leaves number 2 and 4 (from the bottom up) in plants with seven leaves. Asterisks denote significant difference (Student’s *t* test; *P<0.05, **P<0.01, ***P<0.0001). (D) Abaxial epidermal tissues from leaf number 2 (mature leaf) and 4 (young leaf) of M82 and *35S>>CKX3* plants. Bars=50 µm. (This figure is available in colour at *JXB* online.)

The retarded growth of the *35S>>CKX3* plants (Supplementary Fig. S1B) was probably a major cause of the lower whole-plant transpiration (expressed as relative weight change in Fig. 1C). Nevertheless, even after normalizing the daily transpiration rate to leaf area to eliminate the effect of plant size, we still found significantly lower transpiration in the *35S>>CKX3* plants (Fig. 1A). Thus, factors in addition to plant size probably contributed to the reduced transpiration rate. We did not find differences between M82 and *35S>>CKX3* plants in stomatal aperture (Fig. 3A), but stomatal density (the number of stomata per given leaf area; Figs 3B and 4D) was significantly reduced in *35S>>CKX3* plants, due to either a general effect of CK on epidermal cell division or a specific effect of CK on stomatal patterning. To determine which of these possibilities was the case, we calculated the stomatal index (the ratio between stomata and epidermal pavement cells in a given leaf area). The *35S>>CKX3* leaves had fewer and larger pavement cells and fewer stomata (per unit leaf area) than the M82 leaves, but the ratio was not affected by the transgene (Fig. 3C). This suggested that CK deficiency affects epidermal cell division but not stomatal patterning. Since *35S>>CKX3* plants had smaller leaves with reduced stomatal density, the total number of stomata per transgenic plant is expected to be much smaller than that in M82 control plants. Taken together, the results from both experiments indicated that changes in CK levels affect plant transpiration indirectly through developmental mechanisms and not through changes in stomatal movement.

Mature *35S>>CKX3* leaves began to senesce and lose chlorophyll earlier than M82 leaves of the same age (Fig. 4A, Supplementary Fig. S4). Stomatal opening has been shown to be suppressed in senescing leaves (Wardle and Short, 1983). We therefore examined stomatal aperture in leaves number 2 (older) and 4 (younger) in transgenic and M82 plants (both at the seven-leaf stage). In the younger leaves (leaf number 4), we found no differences in stomatal aperture between *35S>>CKX3* and M82 plants (Fig. 4B, D). In contrast, the older *35S>>CKX3* leaves (leaf number 2) displayed reduced stomatal aperture and transpiration rate relative to the older M82 leaves (Fig. 4B, C).

Reduced photosynthetic activity in senescing leaves has been suggested to increase CO_2_ levels, leading to stomatal closure (Thimann and Satler, 1979*a*, b; Wardle and Short, 1983; Willmer *et al.*, 1988). To examine this possibility, we peeled abaxial epidermal tissues from older leaves (leaf number 2) of M82 and *35S>>CKX3* plants and analyzed stomatal opening after incubation in opening solution. Stomatal opening (aperture) was similar in the M82 and *35S>>CKX3* leaves (Supplementary Fig. S5), suggesting that when *35S>>CKX3* stomata are separated from the mesophyll tissue, they open normally. These results supported the hypothesis that reduced CK levels in *35S>>CKX3* leaves promote stomatal closure indirectly. It is possible that the accelerated senescence reduces photosynthesis and increases CO_2_ levels, which in turn promotes stomatal closure. This stomatal closure in the older *35S>>CKX3* leaves probably contributed to the observed reduced whole-plant transpiration.

**Fig. 5.**
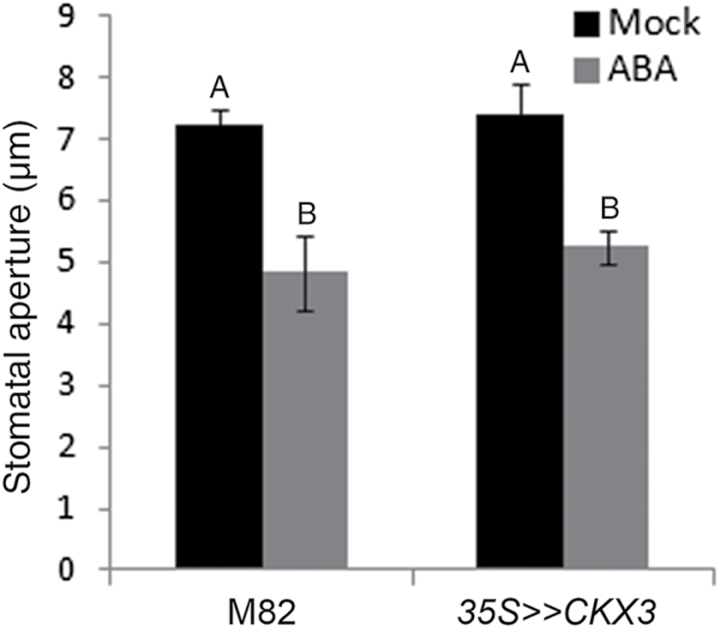
Effect of abscisic acid (ABA) on *35S>>CKX3* and M82 stomatal apertures in tomato. Ten-week-old M82 and *35S>>CKX3* plants were sprayed with 100 µM ABA or water (Mock); 1.5 h later, epidermal imprints were taken from leaf number 2 (counted from the apex of the plant down). Values are means±SE of six replicates, each with ~100 measurements (stomata). Different letters above the columns represent significant differences between treatments (Tukey–Kramer HSD; *P*<0.05).

The role of CK in the plant drought response has been linked to its antagonistic interaction with ABA (Nishiyama *et al.*, 2011; Pospíšilová, 2003*a*; Tanaka *et al.*, 2006). To determine whether reduced CK levels affect the stomatal response to ABA, we analyzed stomatal aperture in ABA-treated *35S>>CKX3* and M82 leaves. ABA had a similar effect on stomatal closure in the two genetic backgrounds (Fig. 5).

### CK treatment increases whole-plant transpiration

**Fig. 6.**
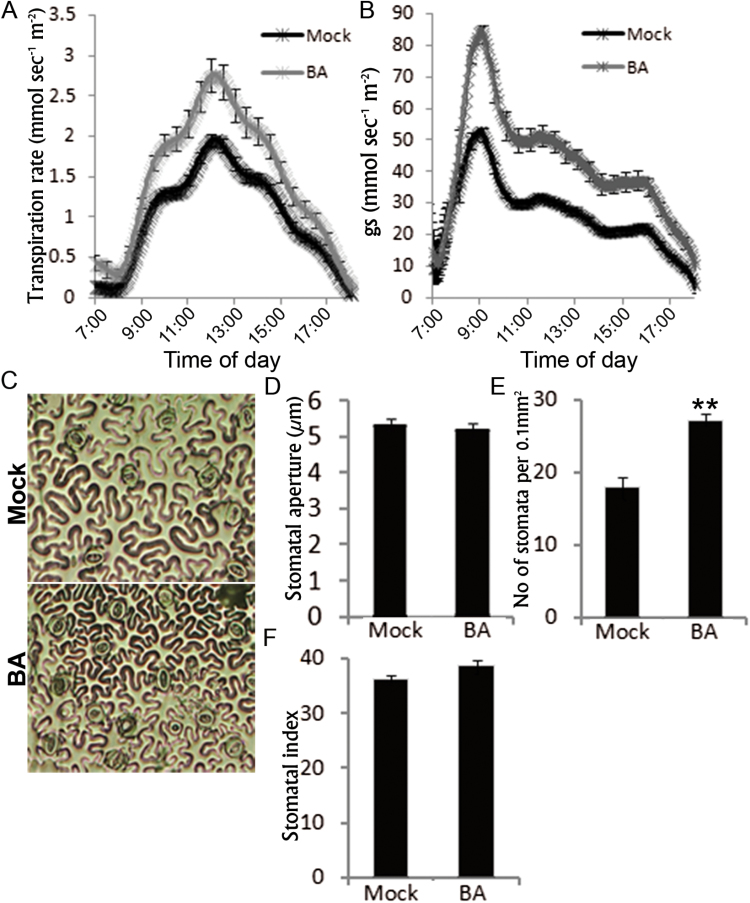
Application of the cytokinin 6-benzylaminopurine (BA) to tomato M82 plants increased plant transpiration and stomatal density. Eight-week-old M82 plants were treated for 7 consecutive days with 100 µM BA or water (Mock). Plants were grown in the greenhouse on lysimeters and daily transpiration was measured 3 days after the last BA treatment. (A) Transpiration rate normalized to total leaf area. (B) Stomatal conductance (*g*_s_) normalized to total leaf area. Values are means±SE of six replicates. (C) Abaxial leaf epidermal tissues of M82 plants treated with BA or water (Mock). Bars=50 µm. (D) Stomatal aperture measured at 10.00 h. (E) Number of stomata per 0.1 mm^2^. (F) Stomatal index (stomata:epidermal cell ratio). For (C) to (F), epidermis imprints were taken from leaf number 3 (from the apex down). In (D) to) (F), values are means±SE of four replicates. In (D), each replicate contained ~100 measurements (stomata). Asterisks denote significant difference (Student’s *t* test; *P*<0.01). (This figure is available in colour at *JXB* online.)

Next, we examined whether the effect of increased levels of CK is the opposite of that of *CKX3* overexpression on plant transpiration. Tomato M82 plants (8 weeks old) were treated with the synthetic CK BA for 7 consecutive days. Three days after the last BA treatment, daily transpiration was measured using an array of load cells (lysimeters). Treated plants displayed a small but significant increase in transpiration rate and stomatal conductance (*g*_s_) (Fig. 6A, B). The daily pattern of stomatal conductance was unaffected (Fig. 6B). This suggested that the BA treatments had no effect on stomatal movement. Microscopic analysis of imprints taken from the abaxial epidermis of young leaves (which unfurled during the treatment) revealed that CK treatment had no effect on stomatal aperture (Fig. 6C, D). This analysis also showed that CK increases stomatal density (the number of stomata per given leaf area; Fig. 6C, E). However, despite finding more and smaller pavement cells and more stomata in a given leaf area, the ratio between stomata and pavement cells was not affected by the BA treatment (Fig. 6F), suggesting that BA has no effect on stomatal patterning. These results suggest that BA increased stomatal density by promoting epidermal cell division. This hypothesis was also supported by increased expression of the cyclin gene *SlCycD3* in BA-treated leaves (Supplementary Fig. S6). It should be noted that while CK treatment increased the total number of cells per leaf area and reduced pavement cell size, it had no effect on the size of the guard cells (Fig. 6C). Since BA treatment had no effect on leaf size or total leaf area (Supplementary Fig. S7), the higher stomatal density should increase the total number of stomata per plant. Taken together, these results suggested that CK promotes whole-plant transpiration by enhancing epidermal cell division, which increases the total number of stomata. The relatively small effect of CK on transpiration (Fig. 6A, B), compared to the strong effect of *CKX3* overexpression, can be attributed to the fact that in this experiment only a small number of leaves were exposed to the BA treatment while they were still in the cell-division phase.

### CK activity in guard cells

The lack of effect of CK on stomatal movement raised the question of whether guard cells are sensitive to this hormone. We examined transgenic tomato expressing the GUS reporter gene under the regulation of the synthetic CK-induced promoter *TCS*v2. *TCSv2* harbors the concatemerized type-B RR-binding motifs (Steiner *et al.*, 2016). Guard cells of the transgenic plants exhibited strong GUS activity following BA treatment (Supplementary Fig. S8), suggesting that guard cells are sensitive to CK.

This strong response in the guard cells raised the possibility that CK has a rapid but transient effect on stomatal movement that cannot be detected after several days. To examine this possibility, M82 plants were treated with 10 µM BA; 2.5 h later, imprints were taken from the leaf abaxial epidermis and stomatal aperture was analyzed. We did not find any significant effect of the BA treatment on stomatal aperture (Supplementary Fig. S9).

**Fig. 7.**
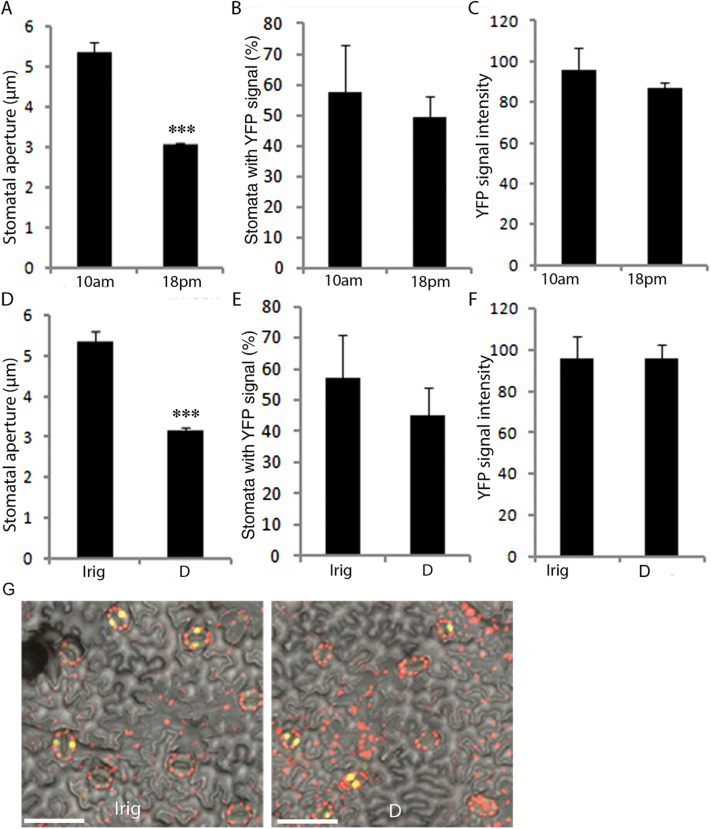
Stomatal aperture and *TCS* activity in guard cells of tomato during the day and in response to drought. (A) Stomatal aperture. (B) Percentage of stomata showing YFP signal. (C) YFP signal intensity. In (A) to (C), measurements were taken at 10.00 h and 18.00 h from *TCSv2:3xVenus (YFP*) leaves. (D) Stomatal aperture. (E) Percentage of stomata with YFP signal. (F) YFP signal intensity. In (D) to (F), measurements were taken at 10.00 h in irrigated (Irig) or mildly drought-stressed (D) *TCSv2:YFP* plants. In (A) to (F), values are means±SE of three replicates. Each replicate is the average of ~50 measurements (stomata). (G) YFP signal in guard cell nuclei of irrigated plants (Irig) and plants exposed to mild drought stress (D). Bars=50µM.

A recent study in Arabidopsis has suggested that CK has a role in the circadian regulation of stomatal movement (Marchadier and Hetherington, 2014). To examine the possible role of CK in daily stomatal movement in tomato leaves, we used transgenic tomato plants expressing the *YFP* reporter gene under the regulation of the *TCSv2* promoter and quantified stomatal aperture and YFP signal in the guard cells, twice a day (morning and afternoon). While the stomata closed toward the afternoon (Fig. 7A), we did not find any changes in the percentage of stomata showing YFP signal, or in the intensity of the YFP signal (Fig. 7B and Supplementary Fig. S10).

It is also possible that transient changes in CK levels play a role in the regulation of stomatal closure in response to water deficiency. We analyzed stomatal aperture and YFP signal in guard cells of *TCSv2:YFP* plants that were either irrigated or exposed to mild drought stress (40–50% VWC). Whereas the drought treatment led to partial stomatal closure (~50%), we did not detect any changes in the YFP signal in guard cells (number of cells showing signals and signal intensity; Fig. 7E–G). Since not all guard cells showed YFP signal, we also compared stomatal aperture in YFP-expressing *versus* non-expressing stomata, in irrigated and drought-treated plants. We did not find differences in stomatal aperture between YFP-expressing and non-expressing stomata, in either treatment (Supplementary Fig. S11). Taken together, we did not find any link between CK activity and stomatal movement in tomato plants.

## Discussion

### CK, transpiration, and stomatal activity

Plants adjust their transpiration rate to changes in the environment through a range of physiological and molecular mechanisms (Osakabe *et al.*, 2014; Yordanov *et al.*, 2000). The rate of transpiration is regulated mainly by stomatal movement but is also affected by stomatal size and density (Jones, 1998; Kramer and Boyer, 1995; Nir *et al.*, 2014). Stomatal movement is regulated by the circadian clock and changes in light conditions, CO_2_ level, temperature, humidity, water availability, and ABA (Farquhar, 1978; Kramer and Boyer, 1995; Pospíšilová, 2003*b*). Previous studies in several plant species have shown that exogenous treatment with CK regulates stomatal aperture (Dodd, 2003; Incoll and Jewer, 1987). In this study, neither endogenous nor exogenous manipulation of CK levels in tomato leaves had any effect on stomatal movement. In addition, we did not find any correlation between the activity of the CK reporter *TCSv2:YFP* in guard cells and stomatal movement. Moreover, CK has been suggested to suppress ABA-induced stomatal closure (Tanaka *et al.*, 2006; Verslues, 2016). In our study, reduced CK levels in *35S>>CKX3* tomato plants had no effect on ABA-induced stomatal closure. Taken together, our results do not support a role for CK in the regulation of diurnal stomatal activity or in the stomatal response to drought or ABA in tomato plants.

However, we did find that stomata in older, but not young, *35S>>CKX3* leaves are relatively closed under conditions that promote stomatal opening. These leaves also exhibited reduced transpiration. Thus, reduced levels of CK promoted stomatal closure in mature leaves. Our results imply that this effect of CK is indirect, probably caused by premature leaf senescence. The effect of CK on leaf senescence is well documented, and transgenic plants with increased CK levels exhibit delayed senescence (Gan and Amasino, 1995). Reduced photosynthesis during senescence (Wingler *et al.*, 1998) has been shown to increase CO_2_ levels in the sub-stomatal compartment, leading to stomatal closure (Thimann and Satler, 1979*a*, b; Wardle and Short, 1983; Willmer *et al.*, 1988). Although previous studies in Arabidopsis and tobacco did not find premature senescence in transgenic plants overexpressing *CKX* (Werner *et al.*, 2003; Macková *et al.*, 2013), in tomato, the *35S>>CKX3* leaves aged prematurely. Thus, it is possible that stomatal closure in mature *35S>>CKX3* leaves was caused by early senescence, which reduced photosynthetic activity in the mesophyll cells and increased CO_2_ levels. Indeed, when we separated stomata of mature *35S>>CKX3* leaves from the mesophyll tissue by peeling abaxial epidermal tissues, we observed normal stomatal opening.

### CK, transpiration, and plant development

Although CK did not affect stomatal movement, it promoted transpiration. Our results suggest that CK affects transpiration by promoting stomatal density through its general effect on cell division (Miller *et al.*, 1956; Schaller *et al.*, 2014). CK treatment of M82 leaves promoted cell division in the abaxial epidermis and, as a result, CK-treated leaves contained more epidermal pavement cells and more stomata, leading to a higher transpiration rate. It should be noted that CK reduced pavement cell size but not guard cell or stomatal pore size. Taken together, these results suggest that increased CK levels increase the transpiration rate indirectly through a general effect on cell division.

Overexpression of *CKX3* strongly reduced whole-plant transpiration. At least three factors therefore affected transpiration in *35S>>CKX3* plants: lower stomatal density, rapid leaf senescence, and reduced leaf size. Leaf size and shape are strongly regulated by environmental conditions and hormones, including CK, and affect transpiration (Bar and Ori, 2014; Fleishon *et al.*, 2011; Pita and Pardos, 2001; Shani *et al.*, 2010). Previous studies have suggested a role for leaf shape and size in plant adaptation to stress conditions (Farris, 1984; Schurr *et al.*, 2000). Under drought conditions, plant and leaf growth are strongly inhibited, in part by increased levels of ABA (Creelman *et al.*, 1990; Zhang and Davies, 1990) and reduced levels of GAs, which lead to accumulation of the growth-suppressor DELLA proteins (Achard *et al.*, 2006; Li *et al.*, 2012; Skirycz and Inzé, 2010). Several studies have shown that CK levels are also reduced under drought conditions (Davies *et al.*, 2005; Murti and Uperti, 2007; Pospíšilová *et al.*, 2000), probably contributing to growth suppression. Thus, the reduction in CK levels may have a role in plants’ natural adaptation to drought by suppressing growth and reducing stomatal density, both of which reduce transpiration. Indeed, previous studies have found reduced stomatal density under drought conditions (Xu and Zhou, 2008). We suggest that drought, CK levels, and stomatal density are linked.

In conclusion, the results of this study show that CK affects tomato plant transpiration by regulating stomatal density and leaf size. We propose that under drought stress, CK levels decrease and, as a result, the rate of cell division is reduced. This suppresses growth and reduces stomatal density, leading to reduced transpiration. The reduced transpiration improves plant survival in drought conditions.

## Supplementary data

Supplementary Table S1. List of primers used in this study.

Supplementary Fig. S1. Transgenic tomato plants overexpressing *AtCKX3*.

Supplementary Fig. S2. Application of the synthetic CK 6-benzylaminopurine (BA) to *CKX3*-overexpressing plants restores normal phenotype.

Supplementary Fig. S3. Overexpression of *AtCKX3* reduces whole-plant transpiration.

Supplementary Fig. S4. *CKX3* overexpression promotes dark-induced senescence.

Supplementary Fig. S5. Stomata of *35S>>CKX3* mature leaves are active.

Supplementary Fig. S6. Exogenous treatment with 6-benzylaminopurine (BA) promotes *CycD3* expression.

Supplementary Fig. S7. Application of CK to M82 plants did not affect plant and leaf size.

Supplementary Fig. S8. CK activates the *TCS* promoter in guard cells.

Supplementary Fig. S9. Effect of exogenous CK treatment on stomatal aperture.

Supplementary Fig. S10. TCS activity in guard cells during the day.

Supplementary Fig. S11. Stomatal aperture of YFP-expressing *versus* non-expressing guard cells under irrigated and drought conditions.

Supplementary Data

## Abbreviations:

ABA: abscisic acid
BA: 6-benzylaminopurine
CK: cytokinin
CKX3: CK oxidase/dehydrogenase 3
GUS: β-galactosidase
GA: gibberellin
IPT: isopentenyl transferase
RR: response regulator
RWC: relative water content
* TCS *: *Two-Component Signaling Sensor*
VPD: vapor-pressure deficit
YFP: yellow fluorescent protein.
