# Supplementary material for: Cytokinin activity increases stomatal density and transpiration rate in tomato
Source: J Exp Bot. 2016 Nov 2;67(22):6351–62. doi: 10.1093/jxb/erw398 (PMC5181579; doi:10.1093/jxb/erw398)
Supplement: Supplementary Data [file supp_67_22_6351__index.html]

Cytokinin activity increases stomatal density and transpiration rate in tomato — Cytokinin activity increases stomatal density and transpiration rate in tomato — Supplementary Data 

# Cytokinin activity increases stomatal density and transpiration rate in tomato

## Supplementary Data

Data files

- Supplementary\_figures\_S1\_S11\_Supplementary\_table\_S1.pdf - Supplementary Data
