# Supplementary material for: Cytokinin activity increases stomatal density and transpiration rate in tomato
Source: J Exp Bot. 2016 Nov 2;67(22):6351–62. doi: 10.1093/jxb/erw398 (PMC5181579; doi:10.1093/jxb/erw398)
Supplement: Supplementary Data [file supp_erw398_Supplementary_figures_S1_S11_Supplementary_table_S1.pdf]

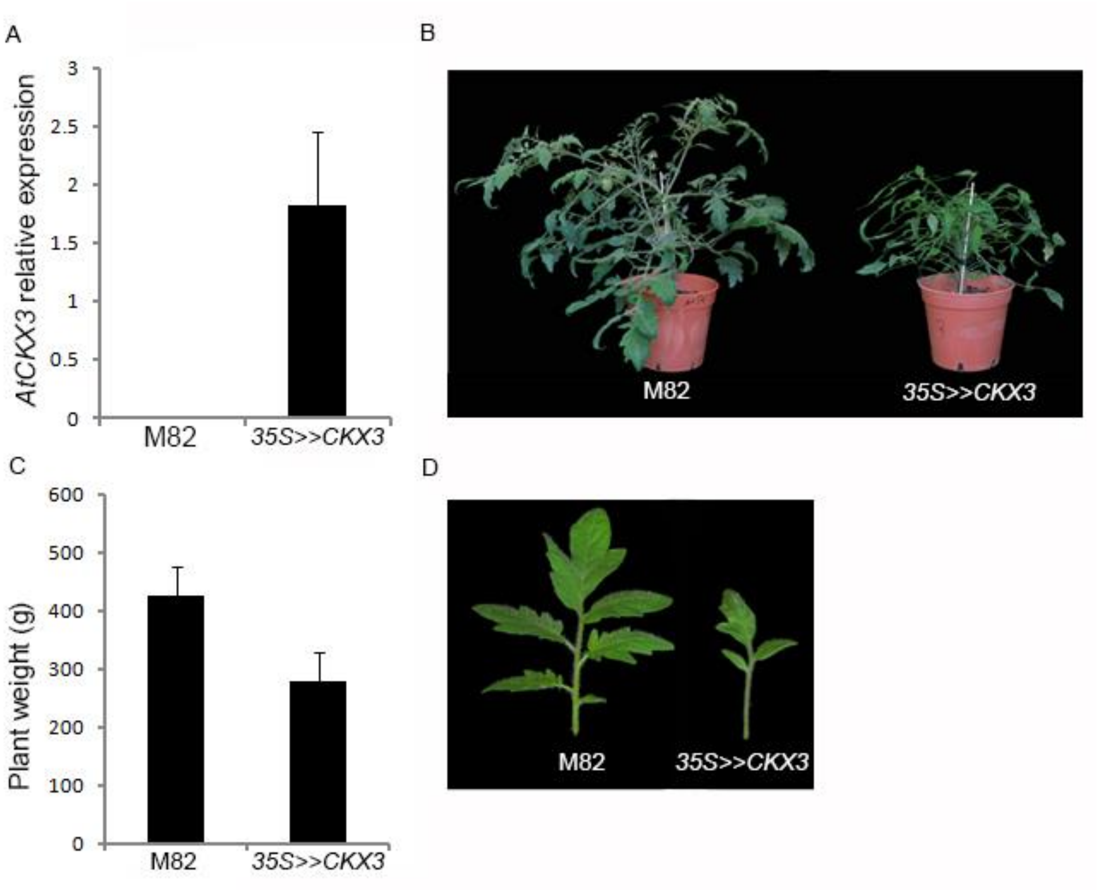

Fig. S1. Transgenic tomato plants overexpressing *AtCKX3*. (A) *AtCKX3* expression in the transactivated leaves. The results are average of three biological replicates ± SE. (B) Representative control M82 and transgenic *35S>>CKX3* plants. (C) Average weight of 2-month-old M82 and *35S>>CKX3* plants. Values are means of five biological replicates ± SE. (D) Leaf no. 3 of 3-week-old M82 and transgenic plants.

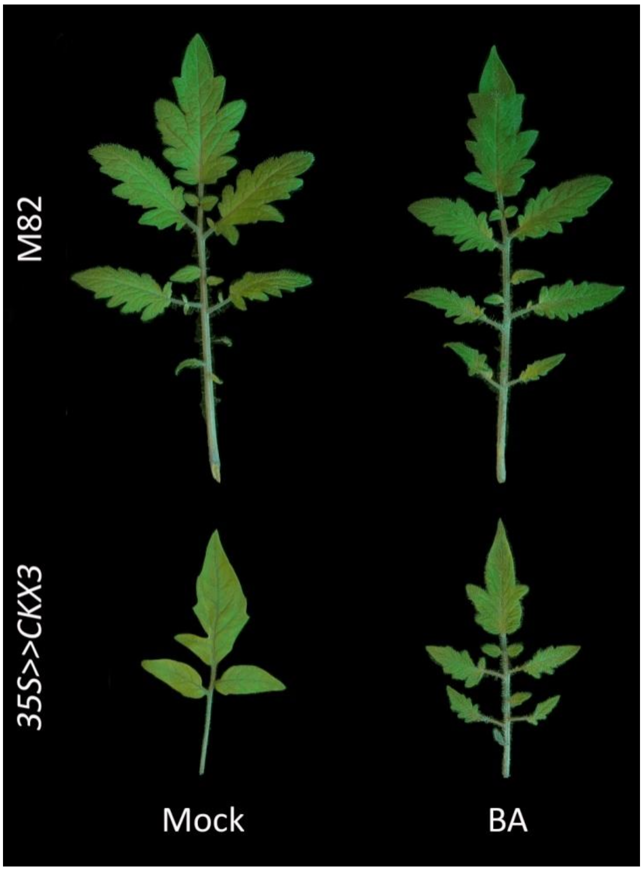

Fig. S2. Application of the synthetic cytokinin 6-benzylaminopurine (BA) to *CKX3*-overexpressing plants restores normal phenotype. M82 and *35S>>CKX3* plants with two true leaves were sprayed with 10 µM BA or water (Mock) for 10 days, after which they were photographed.

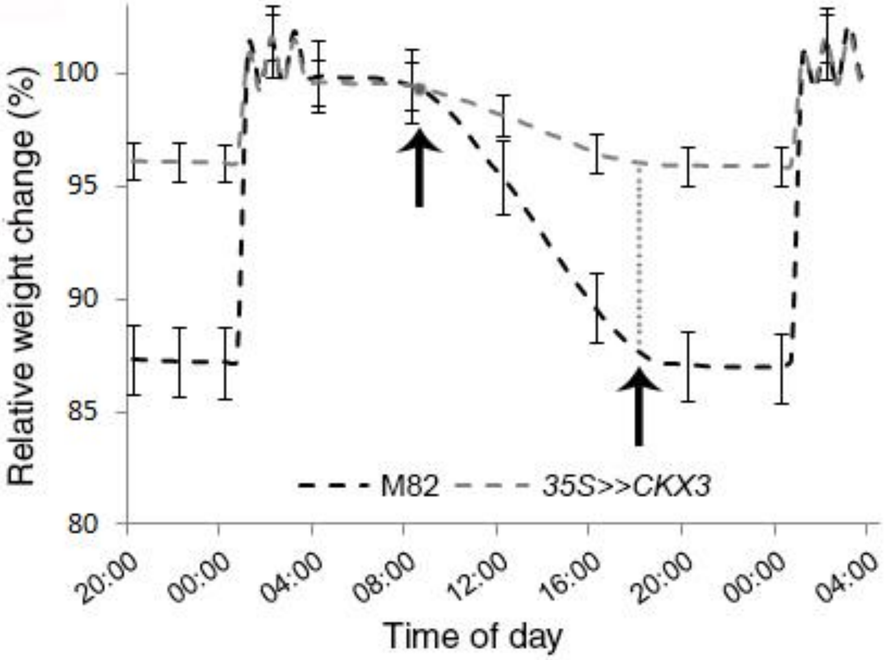

Fig. S3. Overexpression of *AtCKX3* reduces whole-plant transpiration. Average variations in pot weight of M82 and *35S>>CKX3* plants during a 24 h cycle. The cycle consisted of double pulse irrigations followed by drainage and the absence of any weight loss during the night; this was followed by weight loss during the day, and the second irrigation pulses. Arrows indicate beginning and end of transpiration, reflecting stomatal opening and closing times, respectively. Values are means of six biological replicates ± SE.

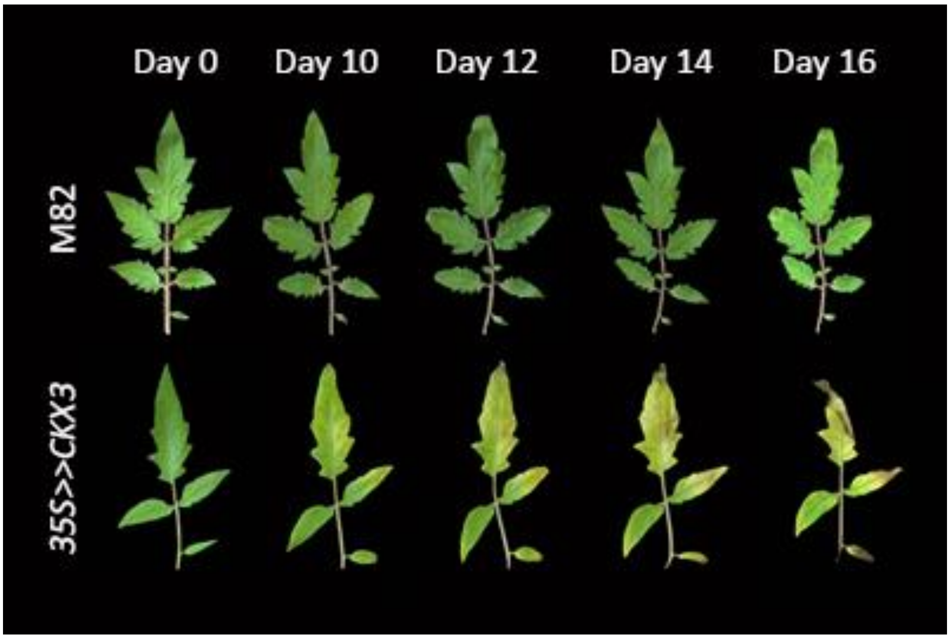

Fig. S4. *CKX3* overexpression promotes dark-induced senescence. Mature leaves of M82 and *35S>>CKX3* were cut and placed in a dark chamber with 100% humidity and 23°C. Photographs were taken after the first leaf showed signs of yellowing (10 days after cutting). Picture shows representative of three biological replicates.

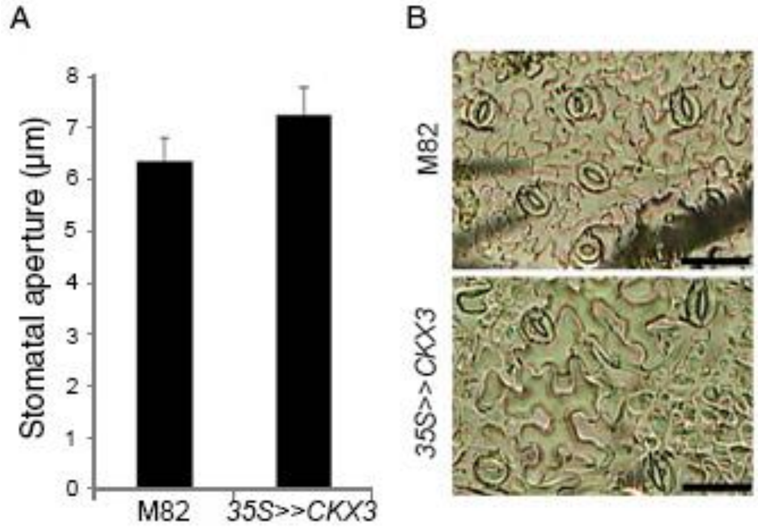

Fig. S5. Stomata of *35S>>CKX3* mature leaves are active. (A) Abaxial epidermis layers were peeled from leaf number 2 (from the bottom up) of M82 and *35S>>CKX3* plants with seven leaves. Epidermal tissues were then immersed in stomatal opening buffer (Wigoda et al., 2006) for 2 h after which the peels were placed on a microscope slide and photographed. Stomatal images were analyzed to determine aperture size using ImageJ software. Values are means of ca. 50 measurements (stomata) in three replicates ± SE. (B) Abaxial epidermal tissues from leaf number 2 of M82 and *35S>>CKX3* plants. Bars = 50 µm.

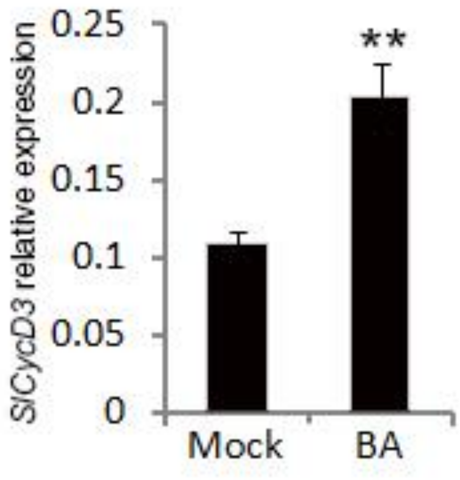

Fig. S6. Exogenous treatment with 6-benzylaminopurine (BA) promotes *CycD3* expression. Expression level (qRT-PCR) of *SlCycD3* in leaf number 3 (from the bottom up) 1.5 h after treatment with 100 µM BA or water (Mock). Values (relative to *TUBULIN*) are means of three biological replicates ± SE. Asterisks denote significant difference (Student's *t* test, $P < 0.01$).

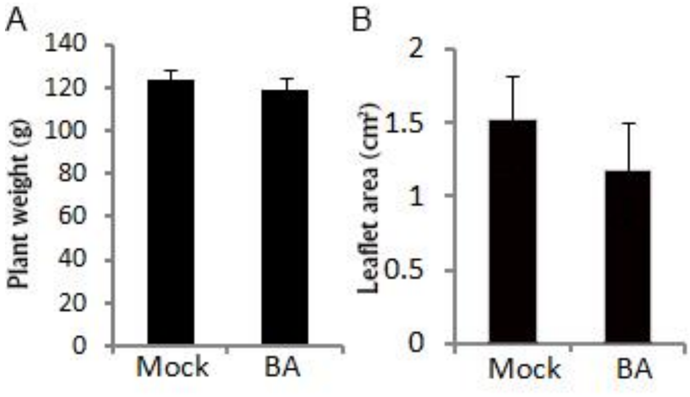

Fig. S7. Application of CK to M82 plants did not affect plant and leaf size. (A) Tomato M82 plants with two true leaves were treated for seven consecutive days with 100 µM 6-benzylaminopurine (BA) or water (Mock). Then plant weight was measured. Values are means of 10 replicates ± SE. (B) Eight-week-old tomato M82 plants were treated for 7 consecutive days with 100 µM BA or water (Mock) and leaflet size (terminal leaflet from leaf number 3) was measured. Values are means of 4 replicates ± standard error (SE).

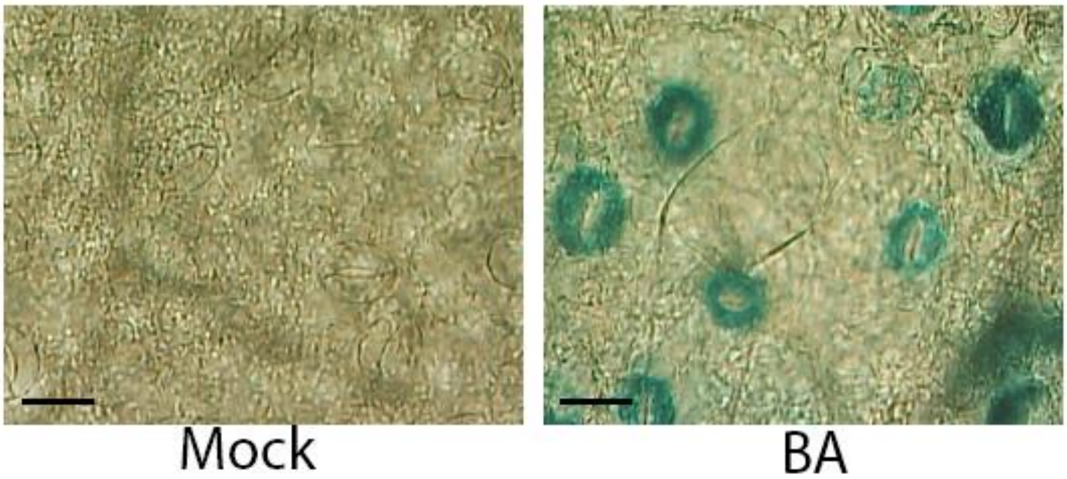

Fig. S8. Cytokinin activates the *TCS* promoter in guard cells. Two-week-old *TCSv2:GUS*-transgenic seedlings were sprayed with 1 μM 6-benzylaminopurine (BA) or water (Mock) for 3 consecutive days; 24 h after the final treatment, histochemical detection of GUS activity in leaf number 1 was performed. Mock and BA-treated tissues were incubated in the staining solution for 14 h. Bar = 20 μm.

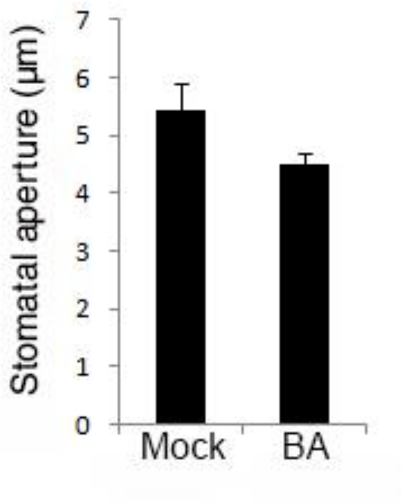

Fig. S9. Effect of exogenous cytokinin treatment on stomatal aperture. Four-week-old M82 plants were sprayed with 10 µM 6-benzylaminopurine (BA) or water (Mock); 2.5 h later, epidermal imprints were taken from leaf number 3 (from the bottom up). The imprints were placed on microscope slides, photographed and then stomatal images were analyzed to determine aperture size. Values are means of four to five replicates, ca. 100 measurements (stomata) each ± SE.

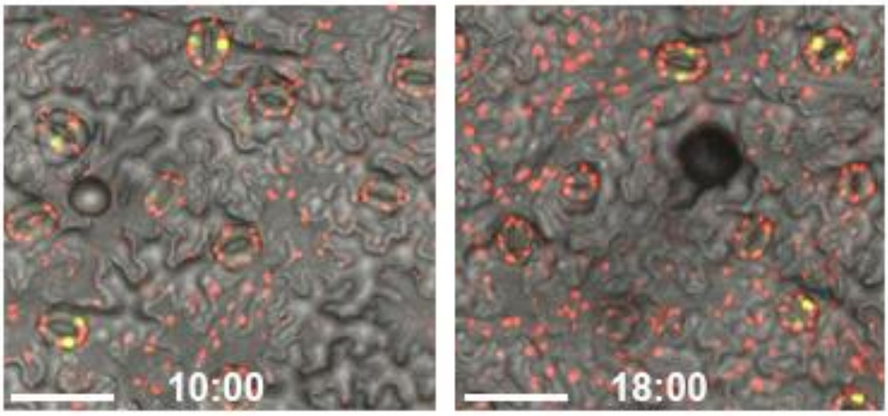

Fig. S10. TCS activity in guard cells during the day. YFP signal in guard cells at 10:00 and 18:00 h in *TCSv2:3xVenus* plants. Bars = 50 µM.

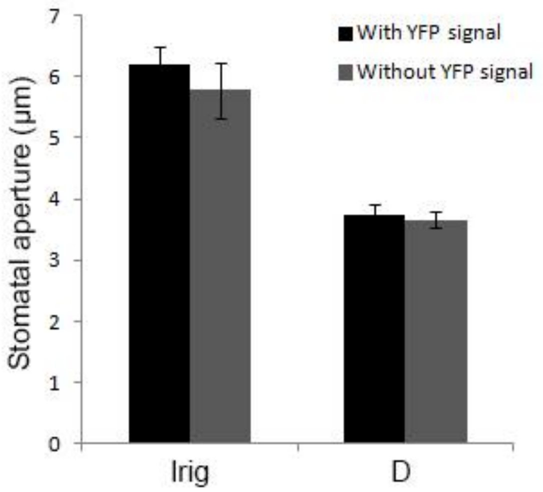

Fig. S11. Stomatal aperture of YFP-expressing vs non-expressing guard cells under irrigated and drought conditions. Stomatal aperture (µm) in irrigated (Irig) or drought stressed *TCSv2:3xVenus* plants (measured separately for YFP-expressing and non-expressing stomata) at 10:00am. Values are means of 3 replicates ± SE. Each replicate ca. 50 measurements (stomata).

Table S1. List of primers used in this study.

Gene	Primers used in this study: 5’→3’[a]
TUBULIN	F- AAACTCACTACCCCCAGCTTT<br>R- GAGAGGAGCAAAACCAACCA
AtCKX3	F-CCAAGGACATGAACTCGGATCT<br>R-GTTTGGGTCAATTCGGCATT
SlCYCD3	F-GCTTAAGCCTTGCATTGGAG<br>R-TCAATGTTGCAAGCCACTTC
GUS	F-ACAGCCAAAAGCCAGACAGA<br>R-TGACGACCAAAGCCAGTAAAG

[a]F, Forward, R, reverse
